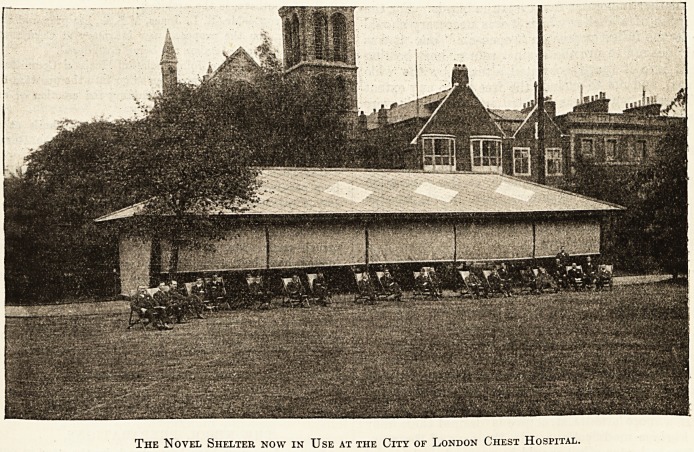# An Original Open-Air Shelter for a Number of Patients Simultaneously

**Published:** 1912-02-10

**Authors:** George Watts

**Affiliations:** Secretary to the City of London Hospital for Diseases of the Chest, E.


### SANATORIA: THEIR USE AND ABUSE.

(Contributions, experiences and questions relating to Sanatoria, their Administration and Work, are invited.)

### An Original Open-Air Shelter for a Number of Patients Simultaneously.

By GEORGE WATTS, Secretary to the City of London Hospital for Diseases of the Chest, E.

The need exists in a considerable number of hospitals for outdoor exercise under cover, or some means of "taking the air" outside the building for patients in a convalescent condition, or having injuries which do not entirely incapacitate them. This need exists in a greater degree in the case of convalescent homes and chest hospitals. The writer is particularly concerned with such provision in an institution of the last-named class, principally regarding consumptive patients. It would appear, however, that the requirements are generally similar, and slight adaptations only are necessary to meet the varying needs of other classes of patients. It is important to draw a distinction between a shelter as a protection to patients in wet or unduly hot weather, and one on the chalet principle, providing sleeping accommodation. The present article deals with the former only.

Assuming that economy is the principal consideration, with due regard to the work being well done, the durability of the shelter, and the reasonable attractiveness of its appearance, the first point for discussion is whether the supports shall be of wood or of iron, and to this point is closely related the question of the flooring. In the case of the shelter for consumptive patients shown in the illustration, erected on the recommendation of the resident medical officer, with special regard to the need of a convenient place for the instruction of the patients in free arm exercises and for recreation under cover when necessary, the requirements as regards flooring were very simple. On each side of the shelter there was already a 12-ft. wide tar-macadam path, and it was decided to lay this material as a floor to the shelter, spreading it to form pathways of 4 ft. width at back and front. In order to obviate discomfort arising from the cold nature of the floor, the patients are provided with foot-rests in the coldest weather. With a floor of this material the least expense arises as regards the fixing of the supports of the shelter.

The Novel Shelter now in Use at the City of London Chest Hospital.

The uprights are of steel, with each base-plate secured in a well-sunk cement concrete bed, rammed solid to the foundation. These stanchions were selected in preference to wood as being more durable and also more suited to the floor adopted. The roof is constructed of timber of substantial kind, with horizontal pieces forming an oblong frame along the tops of the uprights, with similar weight timbers from side to side, and others in the gable of the roof, all being bolted one to another. The roof is covered with yellow deal boarding (treated on the under side with Solignum) and red and grey asbestos slating, the ridge and hips being finished with red tiles and finials at top. The eaves project all round 2 ft. beyond the stanchions, thus providing some protection from wet and heat. Iron gutters are fitted, with swan-necks to carry the rain into the down pipes fastened to the stanchions, and discharge outside the shelter on to the tar paving, which is laid with a camber in order to drain off the water.

A Point for Comment.

In the foregoing description the principal point for comment is the nature of the roof covering. As an alternative to that adopted, corrugated-iron sheeting was considered. The asbestos slating was, however, proved to throw off the sun's rays and so prevent discomfort in hot weather.

Another important feature is the blinds. For the purpose of a shelter which it was necessary should be in the fullest degree an "open-air" one, it was only desired to provide against rain or heat beating right into it (partly obviated already by the wide eaves), and not to exclude the free air to any extent Willesden canvas was selected and the blinds were made half depth, except the two end ones, which in the worst weather afford full protection from wind and rain, and are made of heavy hurricane cloth of the same make as the canvas. In order to keep the blinds taut they were made, at the suggestion of the Hospital's engineer, with heavy wooden rollers (on which they roll up) at bottom, the material being fastened with galvanised tacks to the horizontal timbers above.

The dimensions of the shelter described are 60 ft. by 15 ft., and the cost was as follows:—

| | |
|---|---|
| Flooring (tar paving) ... ... ... | £15 |
| Structure ... ... ... ... ... ... | 107 |
| Blinds ... ... ... ... ... ... | 9 |
| | £131 |

As the shelter described is of novel type, it may be well to state that it has proved entirely satisfactory in extremes both of heat and cold.

If a more artistic structure is desired there may be various modifications. In the case of the stanchions a better effect would be secured with round posts, having decorative pieces where the uprights meet the horizontal frame of the roof; and as regards the roof there are various alternatives, such as tiles of small size or oak shingles.

An important consideration where the shelter is needed for patients not undergoing open-air treatment is the enclosing of the lower part of the sides, and possibly the provision of shutters instead of blinds as a greater protection from wind and rain.

It will be seen that in construction this shelter has something in common with the tents described by Dr. Marrett in The Hospital of January 20, 1912. But the differences are yet considerable, and are to be accounted for by the different design and purpose of the two constructions. The principle underlying both—namely, the need for obtaining the maximum of fresh air and sunshine for pulmonary cases at the minimum of cost—accounts for the similarities. The working out of the details in the two cases is substantially divergent, because one plan is for the permanent residence of a single patient, the other for the occasional or intermittent use of a number of patients simultaneously.

## Figures and Tables

**Figure f1:**